# Prevalence and associated factors of dental implant use in adults and older adults according to self-report and clinical examination: population-based study, SB Brasil 2023

**DOI:** 10.1590/1980-549720260024.supl.1

**Published:** 2026-07-31

**Authors:** Gabriel Schmitt da Cruz, Luiza Souza Schmidt, Yasmim Guterres Bauer, Francisco Wilker Mustafa Gomes Muniz, Ana Lúcia Schaefer Ferreira de Mello

**Affiliations:** I Universidade Federal de Santa Catarina, Graduate Program in Dentistry – Florianópolis (SC), Brazil.; II Universidade Federal de Pelotas, Graduate Program in Dentistry, Department of Semiology and Clinical Practice – Pelotas (RS), Brazil.

**Keywords:** Oral health, Dental health surveys, Dental implants, Aged, Self-report

## Abstract

**Objective:**

To identify factors associated with the use of dental implants in Brazilian adults and older adults.

**Methods:**

This cross-sectional analytical study utilized population-based data from the 2023 National Oral Health Survey. The sample comprised adults (35–44 years) and older adults (65–74 years). Two dichotomous outcomes were modeled: self-reported implant use (yes/no) and clinically observed presence of implants (none/one or more). Adjusted odds ratios (ORs) were estimated using multiple logistic regression for complex samples, with demographic and socioeconomic factors, type of dental service used, and number of missing teeth as independent variables.

**Results:**

The prevalence of dental implants ranged from approximately one-tenth to one-fifth of the sample, depending on the method of identification. In the adjusted analysis for self-reported use, older adults, individuals with higher income, those who used private dental services, and those who sought care for treatment or rehabilitation had significantly higher odds of reporting implant use. Conversely, when analyzing clinically observed implants, only the number of permanent teeth lost showed a significant association.

**Conclusion:**

The use of dental implants in the Brazilian population reflects both the accessibility of dental services and the clinical need for rehabilitation. Using different identification methods provides a more comprehensive understanding of the distribution of implants across the country. These findings can allow the refinement of clinical and organizational criteria for the provision of implant-supported oral rehabilitation within the public system, thereby promoting greater equity and sustainability.

## INTRODUCTION

Oral health is an inseparable and significant component of general health and a marker of inequity^1^. In Brazil, the epidemiological profile of adults and older adults is characterized by a high prevalence of oral diseases that culminate in tooth loss^2^, a significant public health outcome^3–6^.

The consequences of tooth loss extend beyond the oral cavity, impacting stomatognathic functions, social interaction, and quality of life^7–11^. Evidence links tooth loss to adverse systemic outcomes^12^. Thus, a precarious oral condition is both aggravated by and aggravates systemic conditions^8,10,12^.

Data from the latest National Oral Health Survey in Brazil (SB Brasil 2023) confirm the magnitude of the problem: edentulism affects 36.48% of the 65–74 age group, and approximately 70% of older adults and 50% of adults require dental prostheses^13^, a situation that places pressure on the healthcare system beyond the scope of health promotion initiatives or routine care services^8,12,13^.

The placement of implant-supported prostheses represents a treatment option characterized by high survival rates^14,15^, proven efficacy in improving quality of life and masticatory function^16,17^, and potential cost-effectiveness when compared to single crowns or removable prostheses^18–23^. However, access to implants is affected by socioeconomic determinants, creating a scenario of inequity^24^. Factors such as income^25–29^, educational level^26,28^, age^27,28,30^, race/color^25^, use of services^25^, and healthcare financing models^28^ influence the use of implants, favoring individuals with middle to high incomes, higher levels of education, white individuals, and those with recent access to dental care and private insurance coverage, as well as older adults. Income emerges as the most consistent factor, outweighing educational level^27^, thereby highlighting the ability to pay as a universal barrier.

In Brazil, the provision of implants within the Unified Health System (SUS) is governed by the National Oral Health Policy; in 2010, “osseointegrated dental implants” and “implant-supported dental prostheses” were added to the table of procedures, establishing their funding under the Medium and High Complexity categories.

Compared to other interventions, the dental implant is a surgical-prosthetic procedure that entails a higher degree of technological intensity, specialized human resources, and laboratory support services, and carries a higher cost^25,31^. Its implementation within the public health system is limited and concentrated in a few states, resulting in disparities in access^32^. National data from 2011 to 2014 revealed an average of 0.14 implants per 1,000 inhabitants^32^, demonstrating that the policy for implant-supported rehabilitation within SUS has not yet become established as a public health initiative on a large scale^33,34^.

This study was justified by its potential to advance oral health policy within SUS, given that, for the first time, national data are available for epidemiological profiling of rehabilitation with dental implants in Brazil. Therefore, the objective of this study was to identify the demographic, socioeconomic, behavioral, and clinical factors associated with the use of dental implants among adults and older adults in Brazil, based on data from the SB Brasil 2023 Survey.

## METHODS

### Study design, data source, and ethical aspects

A cross-sectional and analytical study was based on secondary data from SB Brasil 2023, a population-based, nationwide epidemiological survey. The report followed the STROBE recommendations^35^.

Anonymized, public-domain data provided by the Ministry of Health were utilized. The original project was approved by the National Research Ethics Commission (Comissão Nacional de Ética em Pesquisa), Opinion No. 4.026.377 (CAAE: 32987320.3.0000.5421).

### Population and sampling

Data from adult participants (aged 35 to 44) and older adults (aged 65 to 74) from the SB Brasil 2023 survey were analyzed. It was a complex and multi-stage sampling design, featuring stratification of primary sampling units and probability proportional-to-size selection.

The total sample for SB Brasil 2023 comprised 48,835 individuals across all age groups. For the purposes of this study, records containing incomplete information regarding the variables of interest were excluded (listwise deletion). The analyses were weighted by sample weights, strata, and clusters to ensure the representativeness of the results relative to the Brazilian population within the investigated age groups.

### Data collection

Data collection was conducted from April to August 2022 by teams (comprising an examining dentist and a dental assistant acting as a recorder) who had undergone training and calibration. Information was obtained through structured interviews — covering demographic and socioeconomic data, service utilization, and clinical oral examinations — in accordance with the criteria and standards of the World Health Organization (WHO)^36^.

The dental prosthesis assessment module included the identification of implant-supported prostheses, single crowns, and partial and complete prostheses (fixed or removable) supported by implants. Examiners were instructed to distinguish these from conventional prostheses based on clinical signs (the presence of metal abutments and the absence of natural roots), without resorting to supplementary examinations. Each implant identified as supporting a prosthesis was recorded individually, including in cases involving overdentures and fixed implant-supported prostheses (protocol prostheses).

Examiner calibration used the *in lux* methodology, based on the analysis of clinical photographs in a virtual environment^37^, and was considered satisfactory when examiners achieved a kappa coefficient of ≥0.61.

### Definition of variables

Outcome 1: Self-reported dental implant: defined based on the question “Do you have any dental implants?”, corresponding to the variable self-reported_implant in the SB Brasil 2023 database. For the analysis, the report of at least one dental implant was considered as presence (yes vs. no).

Outcome 2: Clinical presence of a dental implant: based on the identification of implants during the clinical oral examination, as recorded in the variable “root_implant”, which corresponds to the number of implants clinically detected. For modeling purposes, this variable was dichotomized into absence (0 implants) and presence (≥1 implant).

**Figure 1:**
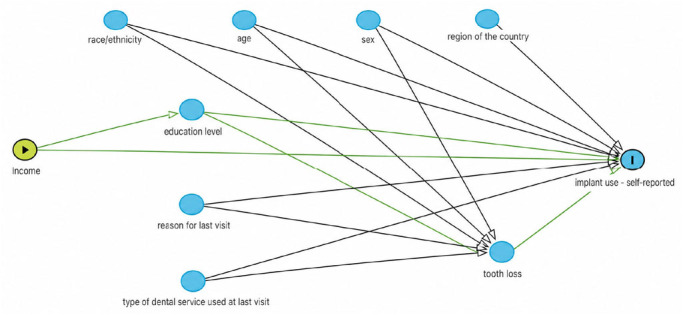
Directed acyclic graph (DAG): variables included in the multivariate analyses for the outcomes “Self-reported implant use” and “Implant use identified by clinical examination”.

The independent variables were grouped into four blocks; details are presented in Chart 1. A theoretical model was constructed based on evidence from previous studies^25–28^ and represented as a directed acyclic graph (DAG), with the aim of guiding the selection of variables included in the multivariate analyses and minimizing confounding biases (Figure 1). It was hypothesized that socioeconomic factors represented by per capita household income and educational level could influence the outcome both directly and indirectly, specifically through oral health conditions, particularly tooth loss. Additionally, demographic variables (age group, sex, color or race, and geographic region) and variables related to dental service utilization (type of service used and reason for the last dental visit) were included, as they were considered potential factors associated with access to and the adoption of rehabilitation treatments.

**Chart 1:** Independent variables considered in the study, method of data collection, and data processing. National Oral Health Survey. (SB Brasil 2023).

Block	Variable (databank name)	SB Brasil 2023 data collection method	Analytical treatment
Demographic	Age group (older adult group)	Derived from age in full years, obtained via household interview.	Recategorized into adults (35–44 years) and older adults (65–74 years); reference category: older adults.
Demographic	Sex (sex)	Participant self-report, recorded as male or female.	Kept as a dichotomous variable; reference category: male sex.
Demographic	Skin color or race (color_race)	Self-report based on the IBGE classification: White, Black, Mixed-race, Asian, and Indigenous.	Kept as five categories; reference category: color/race White.
Demographic	Geographic region (region)	State of residence, subsequently grouped into the five major Brazilian regions (North, Northeast, Southeast, South, and Central-West).	Kept as five categories (reference category: Southeast region).
Socioeconomic	Education (education)	Question: “What is the highest course/grade/school year the participant attended?”, original categories: didn’t attend school; adult literacy; incomplete elementary education; completed elementary education; incomplete high school; completed high school; incomplete higher education; completed higher education; don’t know/didn’t answer.	Recategorized according to the IBGE into four levels: (1) no schooling or incomplete elementary education; (2) complete elementary education or incomplete high school; (3) complete high school or incomplete higher education; (4) complete higher education (reference category). Responses of “don’t know/didn’t answer” were treated as missing data.
Socioeconomic	Per capita household income (percapitaincome)	Calculated according to total monthly household income divided by the number of residents in the household, expressed in reais.	Used as a continuous variable; effect determined per increment of R$ 1,000 in per capita household income.
Use of dental services	Type of dental service used during the last visit (service_type_used)	Question: “Where was your last dental appointment?”, with the categories: never visited a dentist; public service; private practice; health insurance; other	Recategorized into public service and private service or contract (reference category). Participants who never sought care or who responded “other” were excluded from the analyses.
Use of dental services	Reason for the last dentist visit (reason_for_consult_cat)	Question: “What was the main reason for your last visit to the dentist?”, with the categories: preventive, pain, extraction, dental treatment, gum treatment, prosthetics, implants, orthodontics, other.	Recategorized into three categories: preventive or check-up (reference category); pain or urgency; and treatment or rehabilitation. “Don’t know/Didn’t answer” responses and participants who had never sought care were excluded from the analyses.
Clinical	Number of permanent teeth lost (lost)	A clinical examination conducted by trained dentists, using the DMFT index, based on the number of permanent teeth classified as lost.	Used as a continuous variable, representing the total number of permanent teeth lost, with the effect estimated per increment of one lost tooth.

IBGE: Brazilian Institute of Geography and Statistics; DMFT: number of permanent teeth affected by dental caries.

Based on DAG, two multiple logistic regression models were developed, corresponding to the following outcomes: self-reported presence of a dental implant (Model 1) and clinical presence of one or more dental implants (Model 2). In both models, the independent variables included were: age group, sex, color or race, geographic region, type of dental service used at the last visit, reason for the last dental visit, educational level, per capita household income (expressed in reais), and number of permanent teeth lost. The variables per capita household income and number of permanent teeth lost were included as continuous variables, allowing estimation of the variation in the odds ratio associated with a one-unit increment in these measures.

### Statistical analysis

The analyses were conducted using IBM SPSS Statistics 25® software, using the complex samples module and incorporating the weights, strata, and clusters from the sampling design. Modeling was performed identically and independently for each outcome, following a two-stage process:

- Univariate analysis and variable selection: the association between each independent variable and the outcomes was investigated using the Wald F-test for complex samples. All variables defined in the DAG were included in the adjusted models;
- Multivariate modeling: The selected variables were simultaneously included (Enter method) in a multiple logistic regression model for complex samples. Adjusted odds ratios (AOR) with 95% confidence interval (95%CI) were estimated. Associations were considered statistically significant when p<0.05. The overall adequacy of the models was assessed using the Wald F-test. The presence of multicollinearity among the independent variables was evaluated beforehand by calculating the variance inflation factor (VIF) and tolerance.

### Data availability statement

Available Data The dataset supporting the results of this study is publicly available at https://www.gov.br/saude/pt-br/composicao/saps/brasil-sorridente/sb-brasil/dados.

## RESULTS

The final sample consisted of 12,047 participants, where about 70.2% were adults (35–44 years old) and 29.8% were older individuals (65–74 years old). The majority were female (64.6%), and of mixed race/color (40.7%). A gradient of education level was observed among the age groups, with a higher concentration of adults with completed high school or incomplete higher education (46.6%) and of older people with incomplete primary education (57.2%). The use of private or contracted dental services was more frequent among the population studied (57.7%). The average number of permanent teeth lost was 8.03 (95%CI 7.79–8.27), and the average per capita household income was R$ 1,125.64 (95%CI R$ 1,067.21–R$ 1,184.07).

Information regarding other sociodemographic characteristics, service utilization, and oral health status can be found in Table 1.

**Table 1:** Sociodemographic characteristics, service use, and oral health status of the population studied, based on self-report and clinical examination. Brazil, 2020–2023.

Variables	Absence of dental implant (self-report) n=39,684,400; 87.3%	Presence of dental implant (self-report) n=5,754,587; 12.7%	Absence of dental implant (clinical examination) n=36,167,813; 79.0%	Presence of ≥1 dental implant (clinical examination) n=9,638,508; 21.0%
Estimated total (n)	39.684.400	5.754.587	36.167.813	9.638.508
Age group				
Adults (35–44 years)	73.0 (71.1–74.8)	50.9 (45.6–56.2)	71.6 (69.4–73.7)	64.8 (60.9–68.5)
Older adults (65–74 years)	27.0 (25.2–28.9)	49.1 (43.8–54.4)	28.4 (26.3–30.6)	35.2 (31.5–39.1)
Sex				
Male	35.8 (33.8–38.0)	32.0 (26.2–38.4)	35.8 (33.6–38.1)	33.8 (30.0–37.9)
Female	64.2 (62.0–66.2)	68.0 (61.6–73.8)	64.2 (61.9–66.4)	66.2 (62.1–70.0)
Major regions				
North	7.5 (6.2–9.2)	7.0 (4.9–9.8)	7.5 (5.8–9.5)	7.3 (4.7–11.0)
Northeast	27.2 (23.7–31.1)	17.0 (12.7–22.3)	25.5 (21.4–30.0)	27.4 (21.7–33.9)
Southeast	42.8 (37.0–48.8)	49.4 (40.1–58.7)	44.1 (37.3–51.2)	42.7 (34.7–51.2)
South	14.8 (12.2–17.7)	17.7 (13.1–23.3)	16.0 (13.1–19.4)	11.6 (8.1–16.2)
Central-West	7.6 (5.6–10.4)	9.0 (6.4–12.4)	6.9 (5.4–8.9)	11.0 (6.5–18.2)
Color or race				
White	42.9 (39.6–46.3)	53.4 (45.2–61.4)	45.0 (41.6–48.5)	42.1 (35.5–48.9)
Black	13.7 (11.8–15.9)	10.5 (7.4–14.8)	13.3 (11.3–15.5)	13.5 (10.8–16.7)
Asian	1.1 (0.7–1.7)	1.8 (0.9–3.6)	1.1 (0.7–1.5)	1.7 (0.9–3.3)
Mixed-race	42.0 (38.5–45.4)	33.5 (26.6–41.3)	40.3 (36.7–44.0)	42.4 (36.7–48.3)
Indigenous	0.3 (0.1–0.5)	0.7 (0.2–2.2)	0.3 (0.2–0.6)	0.3 (0.1–0.8)
Educational level				
No formal education/incomplete elementary	27.8 (25.3–30.4)	30.6 (24.5–37.3)	27.2 (24.8–29.9)	31.6 (26.4–37.4)
Complete elementary/incomplete high school	19.6 (17.4–22.0)	17.1 (12.4–23.0)	18.9 (16.5–21.6)	20.6 (16.3–25.7)
Complete high school/ incomplete higher education	38.4 (35.3–41.5)	33.5 (26.6–41.2)	38.9 (35.3–42.7)	33.9 (28.9–39.2)
Complete higher education	14.2 (11.9–16.9)	18.9 (14.1–24.7)	14.9 (12.3–18.0)	13.9 (10.4–18.2)
Type of dental service				
Public	44.7 (39.5–50.0)	26.4 (20.1–33.9)	42.3 (36.9–47.8)	42.4 (36.5–48.5)
Private/contract	55.3 (50.0–60.5)	73.6 (66.1–79.9)	57.7 (52.2–63.1)	57.6 (51.5–63.5)
Reason for last dentist visit				
Preventive/routine	29.4 (26.8–32.2)	25.2 (21.8–29.1)	30.3 (27.6–33.1)	22.8 (19.6–26.3)
Pain/urgency	53.4 (50.6–56.3)	42.4 (36.4–48.7)	51.6 (48.4–54.9)	54.6 (49.9–59.3)
Treatment/rehabilitation	17.2 (15.0–19.6)	32.3 (27.2–38.0)	18.1 (15.4–21.0)	22.6 (19.3–26.2)
Continuous variables – Mean (95%CI)				
Per capita household income (R$)	1087.2 (984.4–1190.1)	1226.6 (1075.8–1377.3)	960.0 (831.8–1088.2)	1724.2 (1413.6–2034.9)
Number of permanent teeth lost	7.54 (6.92–8.16)	11.76 (10.2–13.3)	9.98 (8.87–11.09)	10.58 (9.32–11.85)

The self-reported prevalence of dental implants was 9.2% (95%CI 7.1–11.3) among adults and 20.9% (95%CI 16.9–24.9) among older adults. Upon clinical examination, the prevalence of having one or more dental implants was 19.4% (95%CI 15.8–23.0) in adults and 24.8% (95%CI 20.8–28.8) in older adults.

Analysis of the population estimates revealed discrepancies between the methods used to identify dental implants. Clinical assessment estimated that 9,638,508 individuals in Brazil possessed at least one dental implant, whereas self-report identified 5,754,587, corresponding to an absolute difference of 3,883,921 individuals. These results indicate that fewer cases were identified via self-report than through clinical examination.

The sociodemographic profile of individuals with dental implants was similar across both identification methods. For both outcomes, a higher proportion of older adults and a predominance of females were observed. Regionally, the highest concentration of implants occurred in the Southeast, followed by the South and Northeast. Regarding color or race, a higher proportion of individuals self-identifying as White was observed among those with dental implants, regardless of the identification method used.

Socioeconomic and healthcare-related characteristics also varied across the methods. The most frequent level of education among individuals with implants was completion of high school or some college education. The use of private or contracted dental services was more frequent among those with dental implants across both outcomes. Regarding the reason for the last dental visit, a higher frequency of visits for treatment or rehabilitation was observed among individuals with implants. No multicollinearity problems were identified among the independent variables (VIF ranging from 1.02 to 1.77).

### Model 1: factors associated with self-report of dental implants

In multivariate analysis (Table 2), it was observed that age group, per capita household income, type of dental service, and reason for the last dental visit were independently associated with the self-report of dental implants. Adults (35–44 years) showed a lower likelihood of self-reporting dental implants compared to older adults (65–74 years), with OR=0.40 (95%CI 0.23–0.68). Furthermore, an association was observed with per capita household income, showing a 13% increase in the likelihood of self-reporting implants for every R$ 1,000 increment (OR=1.13; 95%CI 1.04–1.22).

**Table 2:** Multivariate analysis: factors associated with self-report of dental implant use. Brazil, 2020–2023.

Variable	Category of comparison	Adjusted OR	95%CI	p-value
Sex	Male	1		0.18
	Female	1.29	0.89–1.89	
Geographic region	Southeast	1		
	North	1.47	0.85–2.55	0.12
	Northeast	0.82	0.47–1.44	0.12
	South	1.18	0.66–2.13	0.12
	Central-West	1.32	0.77–2.26	0.12
Color or race	White	1		
	Black	0.97	0.55–1.73	0.62
	Asian	1.00	0.40–2.47	0.62
	Mixed-race	0.77	0.51–1.18	0.62
	Indigenous	1.72	0.34–8.75	0.62
Type of dental service	Private/contract	**1**		
	Public	**0.60**	**0.39–0.91**	**0.01**
Age group	Older adults (65–74 years)	**1**		
	Adults (35–44 years)	**0.40**	**0.23–0.68**	**0.01**
Reason for dentist visit	Preventive	1		
	Pain/urgency	1.16	0.80–1.67	0.05
	Treatment/rehabilitation	**1.71**	**1.09–2.66**	**0.03**
Educational level	Complete higher education	1		
	No formal education and incomplete elementary	0.66	0.37–1.19	0.19
	Complete elementary and incomplete high school	0.68	0.44–1.04	0.19
	Complete high school and incomplete higher education	0.77	0.52–1.14	0.19
Per capita household income	Increment of R$ 1,000	**1.13**	**1.04–1.22**	**0.01**
Number of permanent teeth lost	Increment of 1 tooth	1.00	0.98–1.02	0.87

95%CI: 95% confidence interval; OR: odds ratio; Estimates in bold indicate statistically significant association (p<0.05).

Individuals treated in the public sector showed a lower likelihood of self-reporting dental implants compared to those treated in private or contracted services (OR=0.60; 95%CI 0.39–0.91). Regarding the reason for the dental visit, individuals who sought care for treatment or rehabilitation showed a higher likelihood of self-reporting dental implants compared to those who visited for preventive or routine care (OR=1.71; 95%CI 1.09–2.66). No statistically significant association was observed for visits motivated by pain or urgency (OR=1.16; 95%CI 0.80–1.67).

The variables sex, geographic region, color or race, educational level, and number of lost permanent teeth did not show a statistically significant association with self-report of dental implants in the adjusted model.

### Model 2: factors associated with the clinical presence of dental implants

In multivariate analysis (Table 3), a statistically significant association was observed only with the number of lost permanent teeth. For each additional lost permanent tooth, there was a 2% increase in the odds of the presence of at least one dental implant (OR=1.02; 95%CI 1.00–1.03).

**Table 3:** Multivariate analysis: factors associated with the presence of 1 or more dental implants (clinical examination). Brazil, 2020–2023.

Variable	Category of comparison	Adjusted OR	95%CI	p-value
Sex	Male	1		
	Female	1.22	0.95–1.56	0.12
Geographic region	Southeast	1.12	0.51–2.44	0.24
	North			
	Northeast	1.00	0.54–1.83	0.24
	South	0.75	0.37–1.50	0.24
	Central-West	1.80	0.87–3.74	0.24
Color or race	White	1		
	Black	0.87	0.57–1.31	0.87
	Asian	1.03	0.53–1.97	0.87
	Mixed-race	0.86	0.60–1.23	0.87
	Indigenous	1.21	0.40–3.69	0.87
Type of dental service	Private/contract	1		
	public	1.03	0.70–1.53	0.87
Age group	Older adults (65–74 years)	1		
	Adults (35–44 years)	1.02	0.66–1.56	0.95
Reason for dentist visit	Preventive	1		
	Pain/urgency	1.28	0.98–1.68	0.19
	Treatment/rehabilitation	1.31	0.86–1.99	0.19
Education	Complete higher education	1		
	No formal education Incomplete elementary	1.02	0.58–1.81	0.99
	Complete elementary and incomplete high school	0.98	0.56–1.71	0.99
	Complete high school and incomplete higher education	1.01	0.72–1.43	0.99
Per capita household income	Increment of R$ 1,000	1.07	0.98–1.17	0.11
Number of permanent teeth lost	Increment of 1 tooth lost	**1.02**	**1.00–1.03**	**0.02**

95%CI: 95% confidence interval; OR: odds ratio; Estimates in bold indicate statistically significant association (p<0.05).

The variables of sex, geographic region, color or race, type of dental service, age group, reason for the dental visit, education level, and per capita household income showed no statistically significant association with the clinical presence of dental implants in the adjusted model.

## DISCUSSION

Notable contributions of this study include, for the first time, the use of data from a national survey, the incorporation of information on dental implants, and a pioneering approach to analyzing two distinct outcomes, which allowed an investigation into both the care-related and clinical dimensions of this mode of rehabilitation.

A discrepancy was observed between the prevalence rates estimated by the two methods, suggesting that self-report may underestimate the actual prevalence of implants within the population, while clinical examination may overestimate it. This distinction reveals significant contrasts in the distribution pattern of implants within the Brazilian population. This phenomenon may be explained by factors such as a lack of awareness regarding one’s own oral health status, difficulty in distinguishing implants from other types of prostheses, or recall bias. Validation studies of oral health assessment instruments frequently indicate that self-report demonstrates only moderate accuracy regarding conditions that require technical knowledge, as in the case of dental implants, noted in previous studies^38,39^. This finding is crucial for the interpretation of association models, as it indicates that a self-reported outcome carries a component of information and perception that may alter its associations with independent variables.

Other studies have reported implant prevalence rates of 1.98% among North American adults aged 18 and older with at least one missing tooth^28^, 3.1% among older adults (aged 65+) in Japan^26^, 13.3% among adults (aged 20+) in Korea^27^, and 16.7% among older adults in southern Brazil, with an upward temporal trend^27,28^.

Regarding self-reported dental implants, the model variables that showed an association were age group, per capita household income, the reason for the most recent dental visit, and the type of service used (public or private). It was observed that older adults (aged 65–74) were more likely to self-report having implants than younger adults; this may reflect a greater accumulation of tooth loss over the course of aging, as well as a heightened need for dental rehabilitation in this demographic^8,12,25^. The use of dental implants in older populations demonstrates satisfactory survival rates and exerts a positive impact on quality of life, provided that adequate follow-up care is maintained^11,15,40^.

Income was positively associated with the self-report of implants, thereby highlighting the sensitivity of this outcome to the socioeconomic gradient. This finding aligns with the existing literature, which has consistently identified income as a determinant in access to implant-based rehabilitation services^25,26,28,30^. Indeed, even individuals with high levels of educational level yet limited income continue to face barriers toaccessing these services^26^.

In Brazil, a socioeconomic gradient is evident in oral health, wherein poorer health outcomes are concentrated among populations characterized by lower income and education levels^31,41^. This disparity is further reflected in patterns of healthcare service use^13,42^. Similarly, the findings of the present study mirror structural patterns regarding access to dental services in Brazil, particularly within the context of SUS. The utilization of dental services is notably higher among individuals with higher income and educational level, as well as among those who pay for their consultations out-of-pocket or possess private dental insurance; this indicates that economic factors exert a profound influence on the use of dental care services throughout the country^42^, despite advances in expanding primary care coverage^43^. The availability of specialized procedures, such as implant-based rehabilitation, remains limited and heterogeneous across municipalities and regions. Furthermore, socioeconomic disparities manifest in higher rates of tooth loss among less-advantaged groups, creating a scenario in which greater clinical need does not always translate into effective access to treatment. Thus, the observed pattern of higher self-reported rates of dental implants among users of private services and higher-income individuals is consistent with evidence indicating that access to higher-cost rehabilitative technologies remains strongly conditioned by structural factors.^26–28^.

Rehabilitation with dental implants is associated with the restoration of masticatory function and an improvement in quality of life, including aesthetic and psychosocial aspects^17,44,45^. In this context, the association between the reason for the last dental visit and self-report implant status suggests that the pursuit of rehabilitation treatment is strongly linked to the perception of functional need and the subjective impact of tooth loss. Evidence indicates that patients seeking implants report significant gains in masticatory comfort and self-esteem, which reinforces the role of subjective experience in the decision-making process regarding treatment^9,45–47^.

In the model regarding the clinical presence of implants, the only variable that remained independently associated was the number of permanent teeth lost. It was observed that for every additional tooth loss, there was a 2% increase in the odds of having at least one dental implant, thereby demonstrating a relationship between cumulative tooth loss and implant-supported rehabilitation. This interpretation is consistent with evidence identifying tooth loss as a central factor in the indication for implant-supported therapies, thereby reinforcing the role of objective rehabilitative need in the actualization of treatment^25,48^.

Internationally, a growing trend in the use of implants is observed, yet socioeconomic and regional inequalities persist^28,49^. The national landscape mirrors this trend: the public provision of dental implants remains restricted and concentrated in a few states, standing in stark contrast to the high prevalence of edentulism and the need for rehabilitation among older people^32–34,50,51^. According to the SUS Outpatient Information System, 196,080 dental implants were placed between 2011 and 2025. Paraná accounts for 62.3% of the total volume, followed by Paraíba with 17.2%, and São Paulo with 7.2%, despite the latter being the most populous state. Amazonas, the Federal District, Acre, Rondônia, Roraima, Tocantins, Maranhão, Sergipe, and Rio Grande do Norte recorded no production during the period analyzed^33^. The limited availability of these implants within SUS, coupled with their concentration in a few states, fosters recourse to legal action as an alternative means of access. While this mechanism ensures care for individual cases, it exacerbates inequity by primarily benefiting groups with higher economic, educational, and legal capital^52^.

According to the SUS procedure fee schedule, the cost of a dental implant is R$ 260.10, to which is added the cost of the implant-supported prosthesis (R$ 300.00), compared to R$ 225.00 for complete or partial removable dentures. Although the initial costs are higher, economic studies demonstrate a superior cost-effectiveness for implants due to their greater longevity, functional stability, and reduced need for replacement^20,21^.

The difference between the models suggests that social determinants^24–27^ primarily influence the care trajectory and the recognition of treatment^52^, whereas the clinical occurrence of implants is more directly related to cumulative tooth loss. This distinction reinforces that the dimensions of need and access operate in a complementary manner regarding the distribution of implants within the Brazilian population^26,42^.

The present study has limitations that should be considered when interpreting the results. As this involves an analysis of secondary data, relevant variables may not have been captured with the desired level of detail, particularly regarding behavioral aspects, access to services, and care trajectories.

Although variable selection was guided by a theoretical model, the simultaneous inclusion of multiple indicators carries a risk of overfitting and may reduce the precision of the estimates. The dichotomization of the clinical presence of implants, by failing to distinguish between different levels of rehabilitative complexity, reduces the analytical sensitivity required to capture treatment gradients.

A discrepancy was observed between the clinical presence of implants and self-reported data, suggesting a potential classification bias. While self-reporting relies on the individual’s ability to accurately recall treatment, thus subject to memory and comprehension biases, the clinical assessment was based exclusively on visual criteria, without radiographic confirmation. The lower estimates derived from self-reporting may be linked to the difficulty in distinguishing implants from other forms of prosthetic rehabilitation. Oral health literacy may influence the ability to recognize complex procedures, particularly given the technical terminology that is frequently confused^53–55^. Finally, sample attrition and potential selection biases may limit the generalizability of these findings to the entire Brazilian population, even though sample weights were incorporated into the analyses.

The findings of this study provide relevant insights to assist public policy-makers in designing, implementing, and evaluating the effective integration of dental implants into SUS, according to clear criteria that integrate both clinical and social aspects. In this context, the importance of strengthening primary care is underscored as a strategy to reduce the incidence of tooth loss and address the social determinants^24^ that shape unequal access to services. This study contributes to the development of a tripartite strategy that is technically sound, financially viable, and committed to equity, ensuring that the expansion of access to high-cost technologies occurs in a manner that effectively reduces oral health inequalities and promotes oral rehabilitation for historically vulnerable groups.

The results obtained inform the enhancement of the oral health care network, guiding access regulation strategies that encompass the optimization of installed capacity, the provision of services, the establishment of clinical protocols, and the development of regulation systems capable of prioritizing patients based on a combination of clinical and socioeconomic criteria.
