# Supplementary material for: Prevalence and associated factors of dental implant use in adults and older adults according to self-report and clinical examination: population-based study, SB Brasil 2023
Source: Rev Bras Epidemiol. 2026 Jul 31;29(Suppl 1):e260024supl1. doi: 10.1590/1980-549720260024.supl.1 (PMC13427312; doi:10.1590/1980-549720260024.supl.1)
Supplement: Translation

# Translation: Prevalência e fatores associados ao uso de implantes dentários em adultos e idosos segundo autorrelato e exame clínico: estudo de base populacional, SB Brasil 2023

**DOI:** 10.1590/1980-549720260024.supl.1.2

**Authors:** Gabriel Schmitt da Cruz, Luiza Souza Schmidt, Yasmim Guterres Bauer, Francisco Wilker Mustafa Gomes Muniz, Ana Lúcia Schaefer Ferreira de Mello

**Affiliations:** I Universidade Federal de Santa Catarina, Programa de Pós-Graduação em Odontologia – Florianópolis (SC), Brasil.; II Universidade Federal de Pelotas, Programa de Pós-graduação em Odontologia, Departamento de Semiologia e Clínica – Pelotas (RS), Brasil.

**Keywords:** Saúde bucal, Inquéritos de saúde bucal, Implantes dentários, Idoso, Autorrelato

## Abstract

**Objetivo:**

Identificar fatores associados a desfechos de reabilitação com implantes dentários em adultos e idosos no Brasil.

**Métodos:**

Estudo transversal, analítico, com dados de base populacional da Pesquisa Nacional de Saúde Bucal, 2023. A amostra incluiu adultos (35–44 anos) e idosos (65–74 anos). Foram modelados dois desfechos dicotômicos: autorrelato de uso de implante (sim/não) e presença clínica de implante (nenhum/um ou mais). As Razões de Chances (RC) ajustadas foram estimadas por meio de regressão logística múltipla para amostras complexas, considerando como variáveis independentes fatores demográficos e socioeconômicos, uso de serviços e número de dentes perdidos.

**Resultados:**

A prevalência de implantes variou entre aproximadamente um décimo e um quinto da amostra, dependendo do método de identificação. Idosos, indivíduos com maior renda, usuários de serviços privados e aqueles que buscaram atendimento por tratamento ou reabilitação apresentaram significativamente maior chance de relatar uso de implante. Na análise da presença clínica, observou-se associação apenas com o número de dentes permanentes perdidos.

**Conclusão:**

O uso de implantes dentários na população brasileira reflete o acesso aos serviços odontológicos e a necessidade clínica de reabilitação. Diferentes formas de identificação ampliam a compreensão sobre o padrão de distribuição dos implantes no país. Os achados oferecem subsídios para o aprimoramento de critérios clínicos e organizacionais para a oferta de reabilitação oral implantossuportada no sistema público, que favoreçam a equidade e a sustentabilidade.

## INTRODUÇÃO

A saúde bucal é um componente indissociável e relevante da saúde geral e um marcador de iniquidade^1^. No Brasil, o perfil epidemiológico de adultos e idosos é marcado pela alta prevalência de doenças bucais que culminam na perda dentária^2^, desfecho relevante de saúde pública^3–6^.

As consequências da perda dentária extrapolam a cavidade bucal, impactando funções estomatognáticas, interação social e qualidade de vida^7–11^. Evidências associam a perda de dentes a desfechos sistêmicos adversos^12^. Assim, a condição bucal precária agrava-se e é agravada por condições sistêmicas^8,10,12^.

Dados do último inquérito nacional sobre a saúde bucal no Brasil (SB Brasil 2023) confirmam a magnitude do problema: o edentulismo atinge 36,48% na faixa etária de 65 a 74 anos, e cerca de 70% dos idosos e 50% dos adultos necessitam de prótese dentária^13^, situação que pressiona o sistema de saúde para além das ações de promoção da saúde ou dos serviços assistenciais^8,12,13^.

A inserção de próteses suportadas por implantes configura uma opção de tratamento com altas taxas de sobrevida^14,15^, comprovada eficácia na melhoria da qualidade de vida e função mastigatória16,17 e potencial custo-efetividade, quando comparada a coroas unitárias ou próteses removíveis^18–23^. Contudo, o acesso a implantes é modulado por determinantes socioeconômicos, configurando cenário de iniquidade^24^. Aspectos como renda^25–29^, escolaridade^26,28^, idade^27,28,30^, raça/cor^25^, utilização de serviços25 e modelo de financiamento em saúde28 influenciam o uso de implantes, favorecendo indivíduos de renda média e alta, com maior escolaridade, brancos, com acesso recente ao atendimento odontológico e cobertos por seguro privado, bem como mais velhos. A renda emerge como o fator mais consistente, superando a escolaridade^27^, o que evidencia a capacidade de pagamento como barreira universal.

No Brasil, a oferta de implantes no Sistema Único de Saúde (SUS) está vinculada à Política Nacional de Saúde Bucal — em 2010, incluiu-se “Implante dentário osseointegrado” e “Prótese dentária sobre implante” na tabela de procedimentos, definindo financiamento na Média e Alta Complexidade.

Comparado aos demais, o implante é um procedimento cirúrgico-protético que incorpora maior densidade tecnológica, recursos humanos especializados e serviços de apoio laboratorial e que tem maior custo^25,31^. Sua realização na rede pública é limitada e concentrada em poucas unidades federativas, resultando em disparidade no acesso^32^. Dados nacionais, entre 2011 e 2014, mostravam uma média de 0,14 implantes por mil habitantes^32^, evidenciando que a política de reabilitação implantossuportada no SUS ainda não se consolidou como uma ação de saúde pública em escala^33,34^.

O presente estudo justifica-se pelo seu potencial de contribuir para o avanço da política de saúde bucal no SUS, uma vez que, pela primeira vez, há dados nacionais que permitem traçar o perfil epidemiológico da reabilitação por meio de implantes dentários no Brasil. Portanto, o presente estudo tem como objetivo identificar os fatores demográficos, socioeconômicos, comportamentais e clínicos associados ao uso de implantes dentários em pessoas adultas e idosas no Brasil, com base em dados do Inquérito SB Brasil 2023.

## MÉTODOS

### Delineamento, fonte de dados e aspectos éticos

Estudo transversal e analítico, baseado em dados secundários do SB Brasil 2023, inquérito epidemiológico de base populacional e de abrangência nacional^13^. O relato seguiu as recomendações STROBE^35^.

Foram utilizados dados anonimizados e de domínio público, disponibilizados pelo Ministério da Saúde. O projeto original foi aprovado pela Comissão Nacional de Ética em Pesquisa, parecer n° 4.026.377 (CAAE: 32987320.3.0000.5421).

### População e amostragem

Foram analisados dados de participantes adultos (de 35 a 44 anos) e idosos (de 65 a 74 anos) do SB Brasil 2023. O plano amostral do inquérito foi complexo, em múltiplos estágios, com estratificação das unidades primárias de amostragem e sorteio com probabilidade proporcional ao tamanho das unidades.

A amostra total do SB Brasil 2023 incluiu 48.835 indivíduos de todas as faixas etárias. Para esse estudo, foram excluídos os registros com informações incompletas nas variáveis de interesse (*listwise deletion*). As análises foram ponderadas pelos pesos amostrais, estratos e conglomerados, a fim de garantir a representatividade dos resultados em relação à população brasileira nas faixas etárias investigadas.

### Coleta de dados

A coleta foi realizada de abril a agosto de 2022 por equipes (um cirurgião-dentista examinador e um auxiliar de saúde bucal anotador) que passaram por treinamento e calibração. As informações foram obtidas por entrevista estruturada — dados demográficos, socioeconômicos, uso de serviços e exame clínico bucal, conforme os critérios e padrões da Organização Mundial da Saúde (OMS)^36^.

O módulo de avaliação de próteses dentárias incluiu a identificação de próteses implanto-suportadas – coroas unitárias, próteses parciais e totais (fixas ou removíveis) apoiadas em implantes. Os examinadores foram instruídos a diferenciá-las de próteses convencionais com base em sinais clínicos (presença de pilares metálicos e ausência de raízes naturais), sem recorrer a exames complementares. Cada implante identificado como suporte de prótese foi anotado individualmente, inclusive em casos de sobredentaduras e de prótese-protocolo.

A calibração dos examinadores utilizou a metodologia *in lux*, baseada na análise de fotografias clínicas em ambiente virtual^37^, considerada satisfatória quando os examinadores atingiram um coeficiente Kappa≥0,61.

### Definição das variáveis

Desfecho 1: Autorrelato de implante dentário: definido a partir da pergunta “O(a) senhor(a) possui algum implante dentário?”, correspondente à variável autorrelato_implante no banco de dados do SB Brasil 2023. Para as análises, foi considerado como presença o relato de pelo menos um implante dentário (sim vs. não).

Desfecho 2: Presença clínica de implante dentário: baseada na identificação de implantes durante o exame clínico bucal, conforme registrado na variável “raiz_implante”, que corresponde ao número de implantes detectados clinicamente. Para fins de modelagem, essa variável foi dicotomizada em ausência (0 implantes) e presença (≥1 implante).

As variáveis independentes foram agrupadas em quatro blocos, cujo detalhamento está no Quadro 1. Foi elaborado um modelo teórico, construído com base em evidências de estudos prévios^25–28^ e representado em gráfico acíclico direcionado (*Directed Acyclic Graph* – DAG), com o objetivo de orientar a seleção das variáveis incluídas nas análises multivariadas e minimizar vieses de confundimento (Figura 1). Considerou-se que fatores socioeconômicos, representados pela renda domiciliar *per capita* e pela escolaridade, poderiam influenciar o desfecho diretamente e também indiretamente, por meio de condições de saúde bucal, particularmente a perda dentária. Adicionalmente, foram incluídas variáveis demográficas (grupo etário, sexo, cor ou raça e região geográfica) e variáveis relacionadas à utilização de serviços odontológicos (tipo de serviço utilizado e motivo da última consulta odontológica), consideradas potenciais fatores associados ao acesso e à adoção de tratamentos reabilitadores.

**Quadro 1:** Variáveis independentes consideradas no estudo, forma de coleta e tratamento. Pesquisa Nacional de Saúde Bucal (SB Brasil 2023).

Bloco	Variável (nome no banco de dados)	Forma de coleta no SB Brasil 2023	Tratamento analítico
Demográfico	Faixa etária (grupo_idosos_adultos)	Derivada da idade em anos completos, obtida por entrevista domiciliar.	Recategorizada em adultos (35–44 anos) e idosos (65–74 anos); categoria de referência: idosos
Demográfico	Sexo (sexo)	Autorrelato do participante, com registro como masculino ou feminino.	Mantida como variável dicotômica; categoria de referência: sexo masculino
Demográfico	Cor ou raça (cor_raca)	Autorrelato conforme classificação do IBGE: branca, preta, parda, amarela e indígena	Mantida com cinco categorias; categoria de referência: cor/raça branca
Demográfico	Região geográfica (regiao)	Unidade federativa de residência, posteriormente agrupada nas cinco grandes regiões brasileiras (Norte, Nordeste, Sudeste, Sul e Centro-Oeste)	Mantida com cinco categorias (categoria de referência: região Sudeste)
Socioeconômico	Escolaridade (escolaridade)	Pergunta: “Qual curso/série/ano escolar mais elevado que o(a) participante frequentou?”, categorias originais: não estudou; alfabetização de adultos; ensino fundamental incompleto; ensino fundamental completo; ensino médio incompleto; ensino médio completo; ensino superior incompleto; ensino superior completo; não sabe/não respondeu	Recategorizada segundo o IBGE em quatro níveis: (1) sem instrução e ensino fundamental incompleto; (2) ensino fundamental completo e ensino médio incompleto; (3) ensino médio completo e ensino superior incompleto; (4) ensino superior completo (categoria de referência). Respostas “não sabe/não respondeu” foram tratadas como dados ausentes.
Socioeconômico	Renda domiciliar *per capita* (rendapercapita)	Calculada a partir da renda familiar mensal total dividida pelo número de moradores do domicílio, expressa em reais.	Utilizada como variável contínua; efeito estimado por incremento de R$ 1.000 na renda domiciliar *per capita*.
Uso de serviços odontológicos	Tipo de serviço odontológico utilizado na última consulta (tipo_servico_utilizado)	Pergunta: “Onde foi sua última consulta odontológica?”, com as categorias: nunca foi ao dentista; serviço público; serviço particular; plano de saúde ou convênio; outros.	Recategorizada em serviço público e serviço privado ou convênio (categoria de referência). Participantes que nunca consultaram ou responderam “outros” foram excluídos das análises.
Uso de serviços odontológicos	Motivo da última consulta odontológica (motivo_da_consulta_cat)	Pergunta: “Qual foi o principal motivo da última consulta ao dentista?”, com as categorias prevenção, dor, extração, tratamento dentário, tratamento gengival, prótese, implante, ortodontia, entre outras.	Recategorizada em três categorias: prevenção ou revisão (categoria de referência); dor ou urgência; tratamento ou reabilitação. Respostas “não sabe/não respondeu” e participantes que nunca consultaram foram excluídos das análises.
Clínico	Número de dentes permanentes perdidos (perdido)	Exame clínico realizado por cirurgiões-dentistas treinados, utilizando *o* índice CPO-D, com base no número de dentes permanentes classificados como perdidos.	Utilizada como variável contínua, representando o número total de dentes permanentes perdidos, com efeito estimado por incremento de 1 dente perdido.

IBGE: Instituto Brasileiro de Geografia e Estatística; CPO-D: número de dentes permanentes acometidos pela doença cárie.

**Figura 1:** *Directed Acyclic Graph* (DAG): variáveis incluídas nas análises multivariadas para os desfechos “Uso de implantes autorreferido” e “Uso de implantes identificado por exame clínico”.

Com base no DAG, foram elaborados dois modelos de regressão logística múltipla, correspondentes aos desfechos: autorrelato de presença de implante dentário (Modelo 1) e presença clínica de um ou mais implantes dentários (Modelo 2). Em ambos os modelos, as variáveis independentes incluídas foram: grupo etário, sexo, cor ou raça, região geográfica, tipo de serviço odontológico utilizado na última consulta, motivo da última consulta odontológica, escolaridade, renda domiciliar *per capita*, expressa em reais, e número de dentes permanentes perdidos. As variáveis renda domiciliar *per capita* e número de dentes permanentes perdidos foram incluídas como variáveis contínuas, permitindo estimar a variação na razão de chances associada ao incremento unitário dessas medidas.

### Análise estatística

As análises foram conduzidas no *software IBM SPSS Statistics* 25®, utilizando o módulo de Amostras Complexas, incorporando os pesos, estratos e conglomerados do plano amostral. A modelagem foi realizada de forma idêntica e independente para cada desfecho, seguindo duas etapas:

- Análise univariada e seleção de variáveis: investigou-se a associação entre cada variável independente e os desfechos por meio do teste F de Wald para amostras complexas. Todas as variáveis definidas no DAG foram incluídas nos modelos ajustados;
- Modelagem multivariada: as variáveis selecionadas foram incluídas simultaneamente (método Enter) em um modelo de regressão logística múltipla para amostras complexas. Foram estimadas as Razões de Chances Ajustadas (RCA) com intervalos de confiança de 95% (IC95%). As associações foram consideradas estatisticamente significativas quando p<0,05. A adequação global dos modelos foi avaliada pelo teste F de Wald. A presença de multicolinearidade entre as variáveis independentes foi avaliada previamente por meio do cálculo do Fator de Inflação da Variância (FIV) e da tolerância.

### Declaração de disponibilidade de dados

O conjunto de dados que dá suporte aos resultados deste estudo está disponível publicamente e pode ser acessado em https://www.gov.br/saude/pt-br/composicao/saps/brasil-sorridente/sb-brasil/dados.

## RESULTADOS

A amostra final foi composta por 12.047 participantes, dos quais aproximadamente 70,2% eram adultos (35–44 anos) e 29,8% idosos (65–74 anos). A maioria era do sexo feminino (64,6%), de cor/raça parda (40,7%). Observou-se um gradiente de escolaridade entre as faixas etárias, com maior concentração de adultos com ensino médio completo ou superior incompleto (46,6%) e de idosos com ensino fundamental incompleto (57,2%). O uso de serviços odontológicos privados ou conveniados foi mais frequente entre a população estudada (57,7%). A média de dentes permanentes perdidos foi de 8,03 (IC95% 7,79–8,27), e a renda domiciliar *per capita* média foi de R$ 1.125,64 (IC95% R$ 1.067,21–R$ 1.184,07).

As informações sobre as demais características sociodemográficas, de utilização de serviços e de condição de saúde bucal encontram-se na Tabela 1.

**Tabela 1:** Características sociodemográficas, de utilização de serviços e de condição de saúde bucal da população estudada, segundo autorrelato e exame clínico. Brasil, 2020–2023.

Variáveis	Ausência de implante dentário (autorrelato) n=39.684.400; 87,3%	Presença de implante dentário (autorrelato) n=5.754.587; 12,7%	Ausência de implante dentário (exame clínico) n=36.167.813; 79,0%	Presença de ≥1 implante dentário (exame clínico) n=9.638.508; 21,0%
Total estimado (N)	39.684.400	5.754.587	36.167.813	9.638.508
Grupo etário				
Adultos (35–44 anos)	73,0 (71,1–74,8)	50,9 (45,6–56,2)	71,6 (69,4–73,7)	64,8 (60,9–68,5)
Idosos (65–74 anos)	27,0 (25,2–28,9)	49,1 (43,8–54,4)	28,4 (26,3–30,6)	35,2 (31,5–39,1)
Sexo				
Masculino	35,8 (33,8–38,0)	32,0 (26,2–38,4)	35,8 (33,6–38,1)	33,8 (30,0–37,9)
Feminino	64,2 (62,0–66,2)	68,0 (61,6–73,8)	64,2 (61,9–66,4)	66,2 (62,1–70,0)
Grandes regiões				
Norte	7,5 (6,2–9,2)	7,0 (4,9–9,8)	7,5 (5,8–9,5)	7,3 (4,7–11,0)
Nordeste	27,2 (23,7–31,1)	17,0 (12,7–22,3)	25,5 (21,4–30,0)	27,4 (21,7–33,9)
Sudeste	42,8 (37,0–48,8)	49,4 (40,1–58,7)	44,1 (37,3–51,2)	42,7 (34,7–51,2)
Sul	14,8 (12,2–17,7)	17,7 (13,1–23,3)	16,0 (13,1–19,4)	11,6 (8,1–16,2)
Centro-Oeste	7,6 (5,6–10,4)	9,0 (6,4–12,4)	6,9 (5,4–8,9)	11,0 (6,5–18,2)
Cor ou raça				
Branca	42,9 (39,6–46,3)	53,4 (45,2–61,4)	45,0 (41,6–48,5)	42,1 (35,5–48,9)
Preta	13,7 (11,8–15,9)	10,5 (7,4–14,8)	13,3 (11,3–15,5)	13,5 (10,8–16,7)
Amarela	1,1 (0,7–1,7)	1,8 (0,9–3,6)	1,1 (0,7–1,5)	1,7 (0,9–3,3)
Parda	42,0 (38,5–45,4)	33,5 (26,6–41,3)	40,3 (36,7–44,0)	42,4 (36,7–48,3)
Indígena	0,3 (0,1–0,5)	0,7 (0,2–2,2)	0,3 (0,2–0,6)	0,3 (0,1–0,8)
Escolaridade (nível)				
Sem instrução/fundamental incompleto	27,8 (25,3–30,4)	30,6 (24,5–37,3)	27,2 (24,8–29,9)	31,6 (26,4–37,4)
Fundamental completo/médio incompleto	19,6 (17,4–22,0)	17,1 (12,4–23,0)	18,9 (16,5–21,6)	20,6 (16,3–25,7)
Médio completo/superior incompleto	38,4 (35,3–41,5)	33,5 (26,6–41,2)	38,9 (35,3–42,7)	33,9 (28,9–39,2)
Superior completo	14,2 (11,9–16,9)	18,9 (14,1–24,7)	14,9 (12,3–18,0)	13,9 (10,4–18,2)
Tipo de serviço				
Público	44,7 (39,5–50,0)	26,4 (20,1–33,9)	42,3 (36,9–47,8)	42,4 (36,5–48,5)
Privado/convênio	55,3 (50,0–60,5)	73,6 (66,1–79,9)	57,7 (52,2–63,1)	57,6 (51,5–63,5)
Motivo da consulta				
Prevenção/rotina	29,4 (26,8–32,2)	25,2 (21,8–29,1)	30,3 (27,6–33,1)	22,8 (19,6–26,3)
Dor/urgência	53,4 (50,6–56,3)	42,4 (36,4–48,7)	51,6 (48,4–54,9)	54,6 (49,9–59,3)
Tratamento/reabilitação	17,2 (15,0–19,6)	32,3 (27,2–38,0)	18,1 (15,4–21,0)	22,6 (19,3–26,2)
Variáveis contínuas – Média (IC95%)				
Renda domiciliar *per capita* (R$)	1087,2 (984,4–1190,1)	1226,6 (1075,8–1377,3)	960,0 (831,8–1088,2)	1724,2 (1413,6–2034,9)
Número de dentes permanentes perdidos	7,54 (6,92–8,16)	11,76 (10,2–13,3)	9,98 (8,87–11,09)	10,58 (9,32–11,85)

A prevalência autorreferida de implantes dentários foi de 9,2% (IC95% 7,1–11,3) entre adultos e de 20,9% (IC95% 16,9–24,9) entre idosos. No exame clínico, a prevalência de presença de um ou mais implantes dentários foi de 19,4% (IC95% 15,8–23,0) em adultos e de 24,8% (IC95% 20,8–28,8) em idosos.

A análise das estimativas populacionais revelou discrepâncias entre os métodos de identificação de implantes dentários. A avaliação clínica estimou que 9.638.508 indivíduos possuíam pelo menos um implante dentário no Brasil, enquanto o autorrelato identificou 5.754.587, o que corresponde a uma diferença absoluta de 3.883.921 indivíduos. Esses resultados indicam menos casos identificados por autorrelato do que no exame clínico.

O perfil sociodemográfico dos indivíduos com implantes dentários foi semelhante entre os dois métodos de identificação. Em ambos os desfechos, observou-se maior proporção de idosos e predominância do sexo feminino. Regionalmente, a maior concentração de implantes ocorreu no Sudeste, seguida pelo Sul e pelo Nordeste. Em relação à cor ou raça, observou-se maior proporção de indivíduos autodeclarados brancos entre aqueles com implantes dentários, independentemente do método de identificação.

As características socioeconômicas e assistenciais também apresentaram variações entre os métodos. O nível de escolaridade mais frequente entre os indivíduos com implantes foi o ensino médio completo ou superior incompleto. A utilização de serviços odontológicos privados ou conveniados foi mais frequente entre aqueles com implantes dentários em ambos os desfechos. Quanto ao motivo da última consulta odontológica, observou-se maior frequência de consultas por tratamento ou reabilitação entre os indivíduos com implantes.

Não foram identificados problemas de multicolinearidade entre as variáveis independentes (FIV variando de 1,02 a 1,77).

### Modelo 1: Fatores associados ao autorrelato de implante dentário

Na análise multivariada (Tabela 2), observou-se que o grupo etário, a renda domiciliar *per capita*, o tipo de serviço odontológico e o motivo da última consulta odontológica estiveram associados, de forma independente, ao autorrelato de implantes dentários. Adultos (35–44 anos) apresentaram menor chance de autorrelatar implantes dentários em comparação aos idosos (65–74 anos), considerando-se RC=0,40; IC95% 0,23–0,68. Observou-se, ainda, associação com a renda domiciliar *per capita*, com aumento de 13% na chance de autorrelato de implantes para cada incremento de R$ 1.000 (RC=1,13; IC95% 1,04–1,22).

**Tabela 2:** Análise multivariada: fatores associados ao autorrelato de uso de implantes dentários. Brasil, 2020–2023.

Variável	Categoria de comparação	RC ajustada	IC95%	p-valor
Sexo	Masculino	1		0,18
	Feminino	1,29	0,89–1,89	
Região geográfica	Sudeste	1		
	Norte	1,47	0,85–2,55	0,12
	Nordeste	0,82	0,47–1,44	0,12
	Sul	1,18	0,66–2,13	0,12
	Centro-Oeste	1,32	0,77–2,26	0,12
Cor ou raça	Branca	1		
	Preta	0,97	0,55–1,73	0,62
	Amarela	1,00	0,40–2,47	0,62
	Parda	0,77	0,51–1,18	0,62
	Indígena	1,72	0,34–8,75	0,62
Tipo de serviço odontológico	Privado/convênio	**1**		
	Público	**0,60**	**0,39–0,91**	**0,01**
Grupo etário	Idosos (65–74 anos)	**1**		
	Adultos (35–44 anos)	**0,40**	**0,23–0,68**	**0,01**
Motivo da consulta odontológica	Prevenção	1		
	Dor/urgência	1,16	0,80–1,67	0,05
	Tratamento/reabilitação	**1,71**	**1,09–2,66**	**0,03**
Escolaridade	Superior completo	1		
	Sem instrução e fundamental incompleto	0,66	0,37–1,19	0,19
	Fundamental completo e médio incompleto	0,68	0,44–1,04	0,19
	Médio completo e superior incompleto	0,77	0,52–1,14	0,19
Renda domiciliar per capita	Incremento de R$ 1.000	**1,13**	**1,04–1,22**	**0,01**
Número de dentes permanentes perdidos	Incremento de 1 dente	1,00	0,98–1,02	0,87

IC95%: Intervalo de Confiança de 95%; RC: razão de chance; Estimativas em negrito indicam associação estatisticamente significativa (p<0,05).

Indivíduos atendidos no serviço público apresentaram menor chance de autorrelatar implantes dentários em comparação aos atendidos em serviços privados ou conveniados (RC=0,60; IC95% 0,39–0,91). Quanto ao motivo da consulta odontológica, indivíduos que buscaram atendimento por tratamento ou reabilitação apresentaram maior chance de autorrelatar implantes dentários em comparação àqueles que realizaram consultas por prevenção ou rotina (RC=1,71; IC95% 1,09–2,66). Não foi observada associação estatisticamente significativa para consultas motivadas por dor ou urgência (RC=1,16; IC95%:0,80–1,67).

As variáveis sexo, região geográfica, cor ou raça, escolaridade e número de dentes permanentes perdidos não apresentaram associação estatisticamente significativa com o autorrelato de implantes dentários no modelo ajustado.

### Modelo 2: Fatores associados à presença clínica de implante dentário

Na análise multivariada (Tabela 3), observou-se associação estatisticamente significativa apenas com o número de dentes permanentes perdidos. Para cada dente permanente perdido adicional, houve aumento de 2% na chance de presença de pelo menos um implante dentário (RC=1,02; IC95% 1,00–1,03).

**Tabela 3:** Análise multivariada: fatores associados à presença de 1 ou mais implantes dentários (exame clínico). Brasil, 2020–2023.

Variável	Categoria de comparação	RC ajustada	IC95%	p-valor
Sexo	Masculino	1		
	Feminino	1,22	0,95–1,56	0,12
Região geográfica	Sudeste	1,12	0,51–2,44	0,24
	Norte			
	Nordeste	1,00	0,54–1,83	0,24
	Sul	0,75	0,37–1,50	0,24
	Centro-Oeste	1,80	0,87–3,74	0,24
Cor ou raça	Branca	1		
	Preta	0,87	0,57–1,31	0,87
	Amarela	1,03	0,53–1,97	0,87
	Parda	0,86	0,60–1,23	0,87
	Indígena	1,21	0,40–3,69	0,87
Tipo de serviço odontológico	Privado/convênio	1		
	público	1,03	0,70–1,53	0,87
Grupo etário	Idosos (65–74 anos)	1		
	Adultos (35–44 anos)	1,02	0,66–1,56	0,95
Motivo da consulta odontológica	Prevenção	1		
	Dor/urgência	1,28	0,98–1,68	0,19
	Tratamento/reabilitação	1,31	0,86–1,99	0,19
Escolaridade	Superior completo	1		
	Sem instrução e ensino fundamental incompleto	1,02	0,58–1,81	0,99
	Fundamental completo e médio incompleto	0,98	0,56–1,71	0,99
	Médio completo e superior incompleto	1,01	0,72–1,43	0,99
Renda domiciliar per capita	Incremento de R$ 1.000	1,07	0,98–1,17	0,11
Número de dentes permanentes perdidos	Incremento de 1 dente perdido	**1,02**	**1,00–1,03**	**0,02**

IC95%: Intervalo de Confiança de 95%; RC: Razão de Chance; Estimativas em negrito indicam associação estatisticamente significativa (p<0,05).

As variáveis sexo, região geográfica, cor ou raça, tipo de serviço odontológico, grupo etário, motivo da consulta odontológica, escolaridade e renda domiciliar per capita não apresentaram associação estatisticamente significativa com a presença clínica de implantes dentários no modelo ajustado.

## DISCUSSÃO

Destacam-se como contribuições do estudo o uso de dados de inquérito nacional, que incorporou, pela primeira vez, informações sobre implantes dentários, e o pioneirismo na análise de dois desfechos, que permitiu investigar dimensões assistenciais e clínicas dessa modalidade de reabilitação.

Observou-se discrepância entre as prevalências estimadas pelos dois métodos, sugerindo que o autorrelato pode subestimar a prevalência real de implantes na população e que o exame clínico pode superestimá-la. Essa diferenciação revela contrastes importantes no padrão de distribuição dos implantes na população brasileira. Esse fenômeno pode ser explicado por fatores como o desconhecimento da própria condição bucal, a dificuldade em distinguir implantes de outros tipos de próteses ou o viés de memória. Estudos de validação de instrumentos de avaliação da saúde bucal frequentemente apontam para acurácia moderada do autorrelato em condições que exigem conhecimento técnico^38,39^, como é o caso dos implantes. Essa constatação é importante para a interpretação dos modelos de associação, pois indica que o desfecho autorreferido carrega um componente de informação e de percepção que pode modificar as associações com as variáveis independentes.

Outros estudos relataram a prevalência de implantes de 1,98% em adultos norte-americanos com 18+ anos com pelo menos 1 dente perdido^28^, 3,1% em idosos 65+ no Japão^26^, 13,3% em adultos 20+ na Coreia^27^, e 16,7% em idosos no Sul do Brasil^25^, com tendência temporal de aumento^27,28^.

Para autorrelato de implantes, as variáveis do modelo que apresentaram associação foram faixa etária, renda domiciliar *per capita*, motivo que levou à última consulta odontológica e o tipo de serviço (público ou privado). Observou-se que pessoas idosas (65–74 anos) apresentaram maior probabilidade de autorrelatar implantes do que adultos, o que pode refletir maior acúmulo de perdas dentárias ao longo do envelhecimento e com a ampliação da necessidade reabilitadora nesse grupo^8,12,25^. O uso de implantes em populações idosas apresenta taxas satisfatórias de sobrevida e impacto positivo na qualidade de vida, desde que haja acompanhamento adequado^11,15,40^.

A renda associou-se positivamente ao autorrelato de implantes, evidenciando a sensibilidade desse desfecho ao gradiente socioeconômico, em consonância com a literatura, que tem apontado consistentemente a renda como fator determinante no acesso às reabilitações com implantes^25,26,28,30^. Mesmo indivíduos com elevada escolaridade, mas baixa renda, enfrentam barreiras no acesso a esses serviços^26^.

No Brasil, observa-se um gradiente socioeconômico na saúde bucal, com piores desfechos concentrados na população de menor renda e escolaridade^31,41^. Essa disparidade também se reflete na utilização de serviços^13,42^. Da mesma forma, os achados desse estudo refletem padrões estruturais de acesso aos serviços odontológicos no Brasil, especialmente no contexto do Sistema Único de Saúde (SUS). O uso de serviços odontológicos é maior entre indivíduos com maior renda e escolaridade e entre aqueles que custeiam consultas ou possuem plano odontológico, indicando que fatores econômicos influenciam fortemente a utilização de cuidados odontológicos no país^42^, apesar dos avanços na ampliação da cobertura da atenção primária^43^. A oferta de procedimentos especializados, como a reabilitação com implantes, permanece limitada e heterogênea entre municípios e regiões. Além disso, as disparidades socioeconômicas se manifestam em maiores taxas de perda dentária entre grupos menos favorecidos, o que configura um cenário em que a maior necessidade clínica nem sempre se traduz em acesso efetivo ao tratamento. Assim, o padrão observado de maior autorrelato de implantes entre usuários de serviços privados e indivíduos de maior renda é coerente com evidências de que o acesso a tecnologias reabilitadoras de maior custo permanece fortemente condicionado por fatores estruturais^26–28^.

A reabilitação com implantes dentários está associada à restauração da função mastigatória e à melhora da qualidade de vida, incluindo questões estéticas e psicossociais^17,44,45^. Nesse contexto, a associação entre o motivo da última consulta e o autorrelato de implantes sugere que a busca por tratamento reabilitador está fortemente vinculada à percepção de necessidade funcional e ao impacto subjetivo da perda dentária. Evidências indicam que pacientes que procuram implantes relatam ganhos relevantes em conforto mastigatório e autoestima, o que reforça o papel da experiência subjetiva na decisão de tratamento^9,45–47^.

No modelo referente à presença clínica de implantes, a única variável que permaneceu associada de forma independente foi o número de dentes permanentes perdidos. Observou-se que, a cada dente adicional perdido, houve um incremento de 2% nas chances de apresentar pelo menos um implante dentário, evidenciando uma relação entre a perda dentária acumulada e a reabilitação implantossuportada. Tal interpretação é consistente com evidências que apontam a perda dentária como evento central na indicação de terapias implantossuportadas, reforçando o papel da necessidade reabilitadora objetiva na realização do tratamento^25,48^.

Internacionalmente, observa-se tendência de crescimento no uso de implantes, mas persistem desigualdades socioeconômicas e regionais^28,49^. O panorama nacional acompanha essa tendência: a oferta pública de implantes dentários continua restrita e concentrada em poucos estados, contrastando com a alta prevalência de edentulismo e a necessidade de reabilitação em idosos^32–34,50,51^. Segundo o Sistema de Informação Ambulatorial do SUS, de 2011 a 2025 foram realizados 196.080 implantes dentários. O Paraná registra 62,3% de toda a produção, seguido pela Paraíba, com 17,2%, e por São Paulo, com 7,2%, apesar de ser o estado mais populoso. Amazonas, Distrito Federal, Acre, Rondônia, Roraima, Tocantins, Maranhão, Sergipe e Rio Grande do Norte não possuem registro de produção no período analisado^33^. A oferta limitada desses implantes no SUS e sua concentração em poucos estados favorecem a judicialização como alternativa de acesso. Tal mecanismo, embora garanta o atendimento a casos individuais, acentua a inequidade ao beneficiar principalmente grupos com maior capital econômico, educacional e jurídico^52^.

O custo de um implante dentário, na tabela de procedimentos do SUS, é de R$ 260,10, acrescido à prótese sobre implante, de R$ 300,00, em comparação a R$ 225,00 para próteses removíveis totais ou parciais. Embora os custos iniciais sejam superiores, estudos econômicos demonstram melhor relação custo-benefício dos implantes devido à maior longevidade, estabilidade funcional e redução da necessidade de substituições^20,21^.

A diferença entre os modelos sugere que determinantes sociais^24–27^ influenciam principalmente a trajetória assistencial e o reconhecimento do tratamento^52^, enquanto a ocorrência clínica do implante está mais diretamente relacionada à perda dentária acumulada. Essa distinção reforça que as dimensões de necessidade e de acesso operam de forma complementar na distribuição dos implantes na população brasileira^26,42^.

O presente estudo apresenta limitações que devem ser consideradas na interpretação dos resultados. Por se tratar de análise de dados secundários, variáveis relevantes podem não ter sido capturadas com o detalhamento desejável, especialmente em aspectos comportamentais, acesso a serviços e trajetórias assistenciais.

Embora a seleção das variáveis tenha sido orientada por modelo teórico, a inclusão simultânea de múltiplos indicadores pode acarretar risco de superajuste e reduzir a precisão das estimativas. A dicotomização da presença clínica de implantes, por não distinguir diferentes níveis de complexidade reabilitadora, reduz a sensibilidade analítica para captar gradientes de tratamento.

Observou-se discrepância entre a presença clínica e o autorrelato de implantes, sugerindo possível viés de classificação. Enquanto o autorrelato depende da capacidade do indivíduo de relatar corretamente o tratamento, estando sujeito a vieses de memória e compreensão, a avaliação clínica baseou-se exclusivamente em critérios visuais, sem confirmação radiográfica. A menor estimativa no autorrelato pode estar relacionada à dificuldade de distinguir implantes de outras formas de reabilitação protética. A literacia em saúde bucal pode influenciar a capacidade de reconhecer procedimentos complexos, especialmente pela terminologia técnica frequentemente confundida^53–55^. Por fim, perdas amostrais e possíveis vieses de seleção podem limitar a generalização dos achados para toda a população brasileira, embora os pesos amostrais tenham sido considerados nas análises.

Os achados deste estudo oferecem subsídios relevantes para que formuladores de políticas públicas desenhem, implementem e avaliem a incorporação eficiente de implantes dentários no SUS, com base em critérios claros que integrem aspectos clínicos e sociais. Nesse contexto, reforça-se a importância de fortalecer a atenção primária como estratégia para reduzir a incidência de perda dentária e enfrentar os determinantes sociais24 que condicionam o acesso desigual aos serviços. O estudo contribui para a construção de estratégia tripartite tecnicamente responsável, financeiramente viável e comprometida com a equidade, assegurando que a ampliação do acesso a tecnologias de alto custo ocorra de modo a reduzir efetivamente as desigualdades em saúde bucal e a promover a reabilitação oral de grupos historicamente vulneráveis.

Os resultados obtidos subsidiam o aprimoramento da rede de atenção à saúde bucal, orientando estratégias de regulação do acesso que incluam a adequação da capacidade instalada, a oferta de serviços, a criação de protocolos clínicos e o desenvolvimento de sistemas de regulação capazes de priorizar pacientes com base em critérios clínicos e socioeconômicos combinados.

## COMITÊ DE ÉTICA:

Foram utilizados exclusivamente dados anonimizados e de domínio público, disponibilizados pelo Ministério da Saúde. O projeto original do inquérito foi aprovado pela Comissão Nacional de Ética em Pesquisa, sob o parecer n° 4.026.377 (CAAE: 32987320.3.0000.5421).
